# Qiu's Neiyi Recipe Regulates the Inflammatory Action of Adenomyosis in Mice via the MAPK Signaling Pathway

**DOI:** 10.1155/2021/9791498

**Published:** 2021-12-11

**Authors:** Pian Ying, Hui Li, Yan Jiang, Zhitao Yao, Shenyi Lu, Huadi Yang, Yingping Zhu

**Affiliations:** ^1^Department of Gynecology and Obstetrics, The First Affiliated Hospital of Zhejiang Chinese Medical University, Hangzhou, Zhejiang 310006, China; ^2^Department of Traditional Chinese Medicine, Pinghu Maternal and Child Health Hospital, PingHu, Zhejiang 314200, China

## Abstract

**Background:**

The management of adenomyosis is challenging and limiting. Qiu's Neiyi recipe (Qiu) is a traditional Chinese medicine (TCM) prescription clinically used for endometriosis treatment in China, but the effect and mechanism of Qiu on adenomyosis are undefined.

**Methods:**

An experimental adenomyosis model was induced in female neonatal ICR mice administrated with tamoxifen. The adenomyosis mice were divided into five groups: high-, middle-, and low-Qiu's group, danazol group, and model group. The mice just administrated with the solvent only (no tamoxifen or drugs) were served as the control group. After 28 days of administration, the body, uterine, spleen, and thymus weights of all mice were examined. Then, the myometrial infiltration and the expression of inflammatory factors were detected by histology examination, ELISA, and qRT-PCR in the uterus. In addition, the MAPK/ERK signaling pathway-related protein expression in adenomyosis mice was detected by immunohistochemical (IHC) staining, qRT-PCR, and western blotting.

**Results:**

In experimental adenomyosis mice, Qiu treatment improved the symptoms of adenomyosis by reducing the myometrial infiltration and increasing the index of spleen and thymus. The elevated levels of IL-1*β*, IL-6, and TNF-*α* in serum and uterus tissues of adenomyosis model mice were also decreased after Qiu treatment. The improvement of Qiu on the adenomyosis was achieved by inhibiting the activated MAPK/ERK signaling pathway, including reducing the mRNA and protein expressions of p-ERK/ERK, p-JNK/JNK, and p-p38/*p*38 in the uterus tissues.

**Conclusion:**

Qiu alleviated the inflammatory reaction and uterus histological changes in mice with adenomyosis, and the potential mechanism is through the inhibition of the MAPK/ERK signaling pathway. Qiu may be a promising treatment for adenomyosis.

## 1. Introduction

Adenomyosis is a common disease occurring in reproductive-aged women. It can cause dysmenorrhea, pelvic pain, and abnormal uterine bleeding, which affects women's health and quality of life [1,2]. Adenomyosis is histologically defined by the invasion of endometrial stroma and glands deeply into the myometrium [3]. It is also associated with the reactive hyperplasia and myometrial hypertrophy induced by chronic inflammatory injuries in the endometrium [4]. Surgery is a surgical treatment commonly used for adenomyosis, accompanied by risks of surgical trauma and infertility [5]. Besides, medication is another important option. Antiestrogen therapeutic drugs, such as danazol and dienogest, are also used to treat adenomyosis clinically [6]. Except for the Western medicine, traditional Chinese medicine (TCM) also has significant advantages on the clinical treatment of adenomyosis, mainly profiting from the distinct curative effect, mild adverse events, and the ability to help patients adjust to menstruation pregnancy [7]. Qiu's Neiyi recipe (Qiu) was founded by the late Professor Qiu Xiaomei and has been used for endometriosis therapy for decades in China [8]. At the same time, its therapeutic effect in endometriosis patients has received traditional Chinese physicians' approval [9,10]. However, the effect and precise molecular mechanism of Qiu on adenomyosis therapy remain unclear and need to be investigated.

A previous study showed that adenomyosis is a kind of chronic inflammatory disease [11]. Various inflammatory cells and inflammatory cytokines accumulate in adenomyosis tissues, and the activated inflammatory response contributes to the process of adenomyosis and related dysmenorrhea and subfertility [12–14]. Meanwhile, the activation of inflammation by related signals is also associated to the inflammatory pathogenesis of adenomyosis [15]. The mitogen-activated protein kinases/extracellular signal-regulated kinases (MAPKs/ERKs) signaling pathway regulates various cellular or intercellular functions, including the growth, differentiation, division, death of cells, and intercellular interaction [16]. In addition, a previous study has found that the MAPK/ERK pathway was activated in endometriosis; ERK, JNK, and p38 belonging to the MAPKs family also play a key role in the inflammatory process and development of adenomyosis [17,18]. Therefore, we suspect that the administration of Qiu might ameliorate the inflammation of adenomyosis through the regulation of the MAPK signaling pathways.

In this study, a model of adenomyosis was established in ICR mice, then the effect and underlying mechanism of Qiu was evaluated on the development of adenomyosis. It was shown that Qiu could reduce the myometrial infiltration of endometrial implants and alleviate the inflammatory response in mice with adenomyosis by suppressing the activated MAPK signaling pathway. The current study may be meaningful for the application of Qiu as a clinical treatment for adenomyosis.

## 2. Materials and Methods

### 2.1. Medicine Preparation

Qiu is composed of the following TCM herbs: 20 g *Sargentodoxa cuneata* (Oliv.) Rehd. et Wils. (Chinese pinyin name: daxueteng), 20 g *LonicerajaponicaThunb* (Chinese pinyin name: rendongteng), 20 g *Spatholobus suberectus* Dunn (Chinese pinyin name: jixueteng), 15 g *Curculigo orchioides* Gaertn. (Chinese pinyin name: xianmao), 15 g *Epimedium brevicornu* Maxim. (Chinese pinyin name: xianlingpi), 12 g *Cistanche salsa* (C. A. Mey.) G. Beck (Chinese pinyin name: congrong), 9 g *Morinda officinalis* How. (Chinese pinyin name: bajitian), 15 g *Taraxacum mongolicum* Hand.-Mazz. (Chinese pinyin name: pugongying), 15 g *Salvia miltiorrhiza* Bge. (Chinese pinyin name: danshen), 10 g *Eupolyphagasinensis Walker* (Chinese pinyin name: dibiechong), and 6 g *Whitmania pigra* Whitman (Chinese pinyin name: shuizhi). All herbs were identified by the pharmacists and provided by the department of pharmaceutical preparation of the First Affiliated Hospital of Zhejiang Chinese Medical University. All the herbs were weighed, mixed, and decocted with 10 folds of water at 100°C to obtain the final concentrations of 0.5 g/mL, 1 g/mL, and 2 g/mL, respectively.

### 2.2. Animals

Newborn ICR mice (day 1 after birth; *n* = 60) and their birth mother mice (*n* = 20) were purchased from the Centre of Experimental Animals at the Shanghai SLAC Laboratory Animal Co., Ltd. (Shanghai, China) with the approval number Certificate No. SCXK (Hu) 2017–0005. All the mice were housed in a standard room temperature of 23 ± 2°C and a humidity of 55–70% under a 12 h light/dark cycle with access to food and water. All animal procedures were in accordance with the National Institutes of Health Guide for the Care and Use of Laboratory Animals. The animal experiments were performed according to the guidelines of laboratory animal care and were authorized by the Ethics Committee of Zhejiang Traditional Chinese Medicine University (Certificate No. SYXK (Zhe) 2018–0012; Hangzhou, China).

### 2.3. Establishment of Adenomyosis Model and Drug Treatment

Female neonatal mice (*n* = 45) were orally administered with 2.7 *μ*mol/kg body weight of tamoxifen suspended in peanut oil/lecithin/condensed milk mixture (2 : 0.2 : 3, by volume) at a dose volume of 5 *μ*L/g body weight from day 2 to day 5 after birth according to the previous study [19]. While female control neonatal mice (*n* = 8) were fed with the equivalent amount of peanut oil/lecithin/condensed milk mixture without tamoxifen. The mice were weaned and separated from their mothers when they were 22 days old. Then, the mice were allowed to eat and drink freely until 3 months. After that, five model mice were randomly selected for pathological examination of uterine tissue, which confirmed the success of adenomyosis modeling.

After that, the mice were successfully molded into 5 groups (8 mice for each group): the model group (water, 0.2 mL), the danazol group (0.02 mg/mL, 0.2 mL), the low dose of the Qiu group (5 g/kg, 0.2 mL), the middle dose of the Qiu group (10 g/kg, 0.2 mL), and the high dose of the Qiu group (20 g/kg, 0.2 mL). At the same time, 8 aforementioned control neonatal mice were treated with solvent only and set as the normal group (water, 0.2 mL). Each group received relative treatment by daily intragastric administration for 28 days.

### 2.4. Detection of the Weight of Immune Organs and Weight Gain

After the treatment, the body weight of each group of mice was recorded. Then, these mice were sacrificed and their uterine, spleen, and thymus were excised. The weight of the uterine, spleen, and thymus were recorded. At the same time, the depth of myometrium invasion was also recorded and scored. The uteruses were postfixed overnight at room temperature in the same fixation solution, then embedded in paraffin for further analysis. The spleen index and thymus index were calculated as follows: spleen index (mg/g) = spleen weight (mg)/body weight (g), thymus index (mg/g) = thymus weight (mg)/body weight (g).

### 2.5. Cytokine Analysis

After weighing the mice, blood was collected from the mice's eyeball and centrifuged at 3,000 rpm for 10 min, following which the supernatant was collected. The levels of IL-1*β*, IL-6, and TNF-*α* in the serum were detected by ELISA according to the manufacturer's instructions in the kit.

### 2.6. Histological Examination

Pathological damage of uterine tissue in each group of mice was detected by hematoxylin and eosin (HE). The uterine tissues were postfixed and embedded in paraffin, then sectioned at a thickness of about 4 *μ*m and stained with HE. HE staining was scored to assess gland hyperplasia based on the following scale [20]: 0 point: normal; 1 point: endometrial glands have small hyperplasia; 2 points: endometrial glands have small hyperplasia, glands or interstitial components invade into the superficial myometrium of the uterus, and a small number of eosinophils appear; 3 points: endometrial glands have large hyperplasia, symptoms of invasion of the superficial myometrium of the uterus by glands or interstitial components increase significantly, and a large number of eosinophils appear; and 4 points: endometrial glands have large hyperplasia, symptoms of invasion of the deep myometrium of the uterus by glands or interstitial components increased significantly, and a large number of eosinophils appear.

### 2.7. Immunohistochemistry

An immunohistochemistry stain was performed to detect the expression of p-ERK, p-JNK, and p-p38 in uterine tissue. In brief, after being deparaffinized and rehydrated, the paraffin-embedded slides were extracted in a 0.1 mol/L citric acid buffer (PH = 6.0) and treated with 3% H_2_O_2_. Then, the slides were incubated with primary antibodies overnight at 4°C. After washing, biotinylated secondary antibodies were added and incubated at room temperature for 20 min. Finally, the sections were stained with DAB to develop the color and counterstained with hematoxylin.

### 2.8. Total RNA Extraction and qRT-PCR

Total RNA was extracted from uterine tissue (myometrium and endometrium) using Trizol reagent (Bioengineering Company Limited, China) according to the manufacturer's instructions. Total RNA was reverse transcribed to cDNA and amplified and analyzed using SYBR Green PCR Master Mix (Kangwei Century Biotechnology Co., Ltd., China) and a CFX96 real-time system (Bio-Rad, USA). Each group was analyzed in triplicate. The original threshold cycle (Ct) values were standardized with *β*-actin by the 2^−ΔΔCt^ method. The primer sequences used are shown in Table 1.

### 2.9. Western Blotting Analysis

After treatment, the uterine tissues (myometrium and endometrium) were lysed with RIPA buffer. After isolation by sodium dodecyl sulfate-polyacrylamide gel electrophoresis (SDS-PAGE), the proteins were transferred to polyvinylidene difluoride (PVDF) membranes. After blocking by 5% defatted milk powder, the membranes were incubated with the primary antibodies against tubulin alpha antibody (AF7010, 1 : 5000, Affinity, USA), ERK1/2 antibody (AF0155, 1 : 1000, Affinity, USA), phospho-ERK1/2 (Thr202/Tyr204) antibody (ab92946, 1 : 500, Affinity, USA), JNK1/2/3 antibody (AF6318, 1 : 500, Affinity, USA), phospho-ERK1/2 (Tyr204) antibody (AF1014, 1 : 500, Affinity, USA), p38 MAPK antibody (AF6456, 1 : 500, Affinity, USA), and phospho-p38 MAPK (Thr180/Tyr182) antibody (AF4001, 1 : 500, Affinity, USA) at 4°C for 24 h. After washing with PBS, the membranes were incubated with the secondary antibodies at room temperature for another 2 h. Lastly, the signals of protein bands were developed with enhanced chemiluminescence (ECL).

### 2.10. Statistical Analysis

The statistical analyses were performed using SPSS 16.0 (IBM, Armonk, NY, USA). Values are expressed as mean + SEM. Group comparisons were processed by using the one-way ANOVA analysis followed by Dunnett's post hoc test according to variance homogeneity and inconsistency. In all cases, *P* < 0.05 was considered as statistical significance.

## 3. Results

### 3.1. Qiu Inhibited the Progression of Adenomyosis Mice

**Figure 1:**
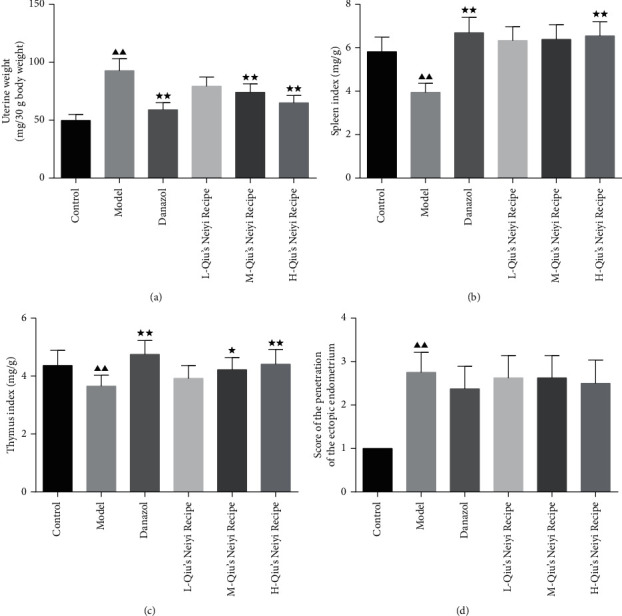
The effect of Qiu's Neiyi recipe on uterine weight, immune organs weight, and gland hyperplasia. (a) The uterine weight of each group of mice. The spleen index (b) and thymus index (c) of each group of mice. (d) Score of the penetration of the ectopic endometrium of each group of mice. L-Qiu's Neiyi recipe: a low dose of Qiu's Neiyi recipe; M-Qiu's Neiyi recipe: a middle dose of Qiu's Neiyi recipe; H-Qiu's Neiyi recipe: a high dose of Qiu's Neiyi recipe. $\left(\bar{\chi }\pm s,n=8\right)$, ΔΔ*P* < 0.01 vs. control group, ^*∗*^*P* < 0.05, ^*∗∗*^*P* < 0.01 vs. model group.

The average food intake for all group mice had no differences. As shown in Figure 1, the uterine weight was significantly increased in both the model and drug treatment groups compared to the control (*P* < 0.01). After being treated with M-/H-Qiu and danazol, the uterine weights significantly decreased (*P* < 0.05). Meanwhile, the mice given a high dose of Qiu treatment had lower uterine weight than the mice given low and middle doses of Qiu treatment. In addition, for the model group, the spleen index and thymus index significantly decreased, with the increased score of penetration of the ectopic endometrium into the midmyometrium compared to the control group (*P* < 0.01). Compared with the model group, after drug treatment, the spleen indexes were significantly increased in the high dose of Qiu and danazol, and the thymus indexes were significantly increased in the middle and high dose of Qiu and danazol (*P* < 0.05). However, the penetration of the ectopic endometrium into myometrium had no obvious improvement.

### 3.2. Qiu Inhibited the Expression of Inflammatory Factors

**Figure 2:**
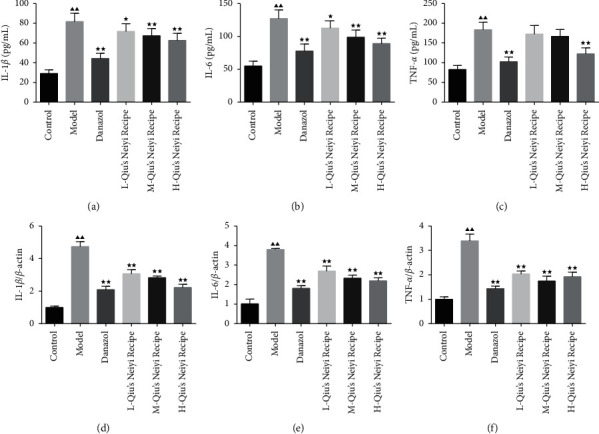
(a–c) Qiu's Neiyi recipe inhibited the expression of IL-1*β*, IL-6, and TNF-*α* in the serum of adenomyosis model mice. (d–f) Qiu's Neiyi recipe inhibited the mRNA expression of IL-1*β*, IL-6, and TNF-*α* in the uterine tissue. L-Qiu's Neiyi recipe: a low dose of Qiu's Neiyi recipe; M-Qiu's Neiyi recipe: a middle dose of Qiu's Neiyi recipe; H-Qiu's Neiyi recipe: a high dose of Qiu's Neiyi recipe. $\left(\bar{\chi }\pm s,n=8\right)$, ΔΔ*P* < 0.01 vs. control group,  ^*∗*^*P* < 0.05,  ^*∗∗*^*P* < 0.01 vs. model group.

The expression levels of inflammation cytokines (IL-1*β*, IL-6, and TNF-*α*) in serum and uterine tissue were measured by ELISA and qRT-PCR. As shown in Figures 2(a)–2(c), after being treated with tamoxifen, three proinflammatory mediators significantly increased compared with those of the control group (*P* < 0.05). The Qiu treatment inhibited the expression of IL-1*β*, IL-6, and TNF-*α* in the tamoxifen-induced adenomyosis mice in a dose-dependent manner. At the same time, the level of these proinflammatory mediators was also decreased after being treated with danazol (*P* < 0.01). In addition, the mRNA expressions of inflammatory factors (IL-1*β*, IL-6, and TNF-*α*) were significantly increased in the model group compared with those of the control group in the uterine tissue (*P* < 0.01). However, danazol and Qiu both suppressed the expression of these inflammatory factors (*P* < 0.01) (Figures 2(d)–2(f)).

### 3.3. Qiu Reduced Gland Hyperplasia and Inflammatory Infiltration in the Uterine Tissue

**Figure 3:**
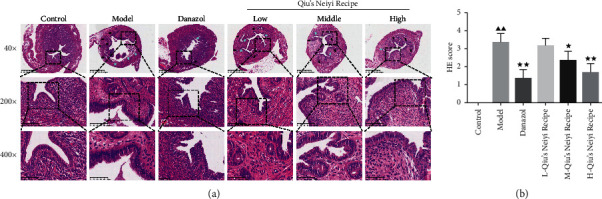
Qiu's Neiyi recipe decreased the hyperplasia of endometrial glands and the invasion of glandular or interstitial components in uterine tissue. (a) Representative microphotographs of HE staining, original magnification 40×, 200×, and 400×. (b) Semiquantitative assessment of the histological lesions. L-Qiu's Neiyi recipe: a low dose of Qiu's Neiyi recipe; M-Qiu's Neiyi recipe: a middle dose of Qiu's Neiyi recipe; H-Qiu's Neiyi recipe: a high dose of Qiu's Neiyi recipe. Yellow and blue arrows indicate the hyperplasia and infiltration locations in HE staining. $\left(\bar{\chi }\pm s,n=6\right)$, ΔΔ*P* < 0.01 vs. control group,  ^*∗*^*P* < 0.05,  ^*∗∗*^*P* < 0.01 vs. model group.

The inflammatory infiltration and penetration of the ectopic endometrium were examined by hematoxylin-eosin (HE) staining. Compared with the control group, there was obviously endometrial glandular hyperplasia and adenomyosis in the myometrium in the model group. The glandular or interstitial components invaded the deep myometrium of the uterus. However, danazol and middle and high doses of Qiu dramatically alleviated this injury (*P* < 0.01). The hyperplasia of endometrial glands decreased, the glandular or interstitial components invaded the superficial myometrium of the uterus, and the structural changes improved significantly compared with the model group (Figure 3).

### 3.4. Effect of Qiu Treatment on the Level of p-ERK, p-JNK, and p-p38 in Mouse Uterine Tissue

**Figure 4:**
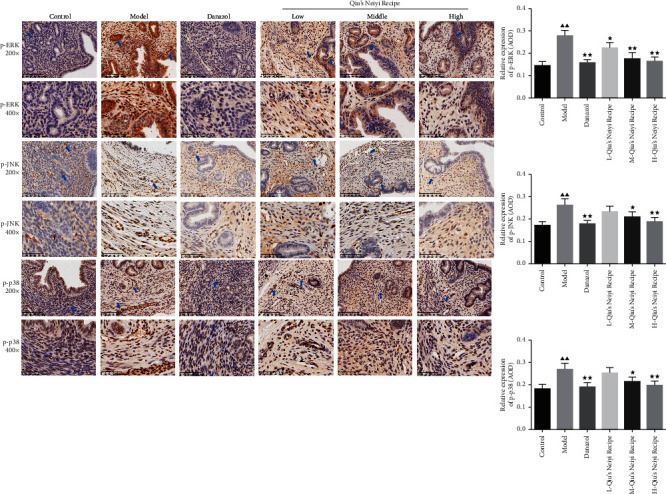
Expressions of p-ERK, p-JNK, and p-p38 in uterine tissue were analyzed by immunochemical staining. (a) Representative microphotographs of immunochemical staining, original magnification, 200× and 400×. (b–d) Quantitative assessment of the expressions of p-ERK, p-JNK, and p-p38. L-Qiu's Neiyi recipe: a low dose of Qiu's Neiyi recipe; M-Qiu's Neiyi recipe: a middle dose of Qiu's Neiyi recipe; H-Qiu's Neiyi recipe: a high dose of Qiu's Neiyi recipe. Blue arrows indicate the target proteins in the immunochemical staining. $\left(\bar{\chi }\pm s,n=6\right)$, ΔΔ*P* < 0.01 vs. control group,  ^*∗*^*P* < 0.05,  ^*∗∗*^*P* < 0.01 vs. model group.

The expressions of p-ERK, p-JNK, and p-p38 in ectopic endometria were detected by immunohistochemistry. As shown in Figure 4, compared with the control group, the expressions of p-ERK, p-JNK, and p-p38 proteins were significantly increased in the model group (*P* < 0.01). However, the expression of these proteins was significantly decreased after being treated with danazol and Qiu (*P* < 0.05). And Qiu notably suppressed their elevated expressions in a dose-dependent manner.

### 3.5. Qiu Suppressed the Activation of the MAPK Signaling Pathway

**Figure 5:**
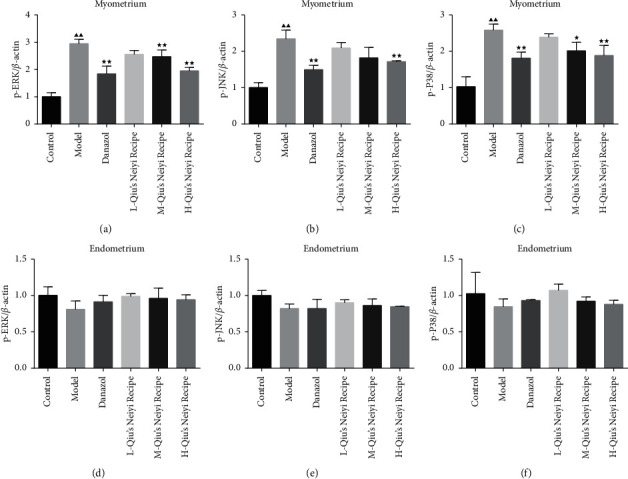
Qiu's Neiyi recipe affects the expression of immune factors and the MAPK signaling pathway-related genes mRNA. (a–c) The expression of p-ERK, p-JNK, and p-p38 in the myometrium. (d–f) The expression of ERK, JNK, and p38 in the endometrium. L-Qiu's Neiyi recipe: a low dose of Qiu's Neiyi recipe; M-Qiu's Neiyi recipe: a middle dose of Qiu's Neiyi recipe; H-Qiu's Neiyi recipe: a high dose of Qiu's Neiyi recipe. $\left(\bar{\chi }\pm s,n=3\right)$, ΔΔ*P* < 0.01 vs. control group,  ^*∗*^*P* < 0.05,  ^*∗∗*^*P* < 0.01 vs. model group.

In order to detect the mechanism of Qiu on the adenomyosis mice, qRT-PCR and western blotting were performed to detect the mRNA and protein expression of MAPK signaling pathway-related genes and proteins. Compared with the control group, the expression of MAPK signaling pathway-related genes (ERK, JNK, and p38) was significantly increased in the myometrium of model group mice (*P* < 0.01). However, the activation of the MAPK signaling pathway was inhibited by danazol and Qiu (*P* < 0.05). And Qiu notably suppressed the expression of these proteins in a dose-dependent manner. Nevertheless, there was no significant difference in the mRNA expression of MAPK signaling pathway-related genes in the endometrium (Figure 5).

**Figure 6:**
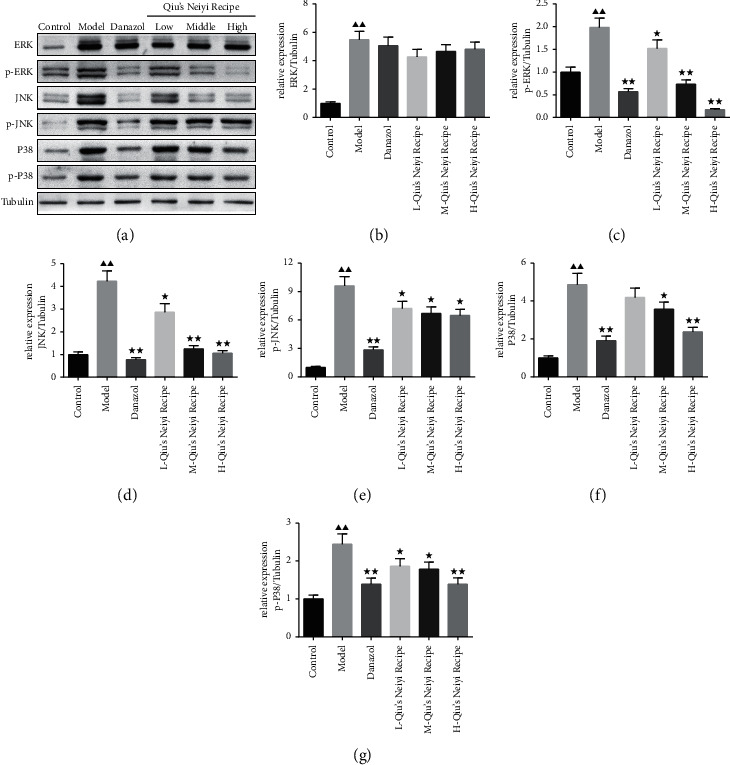
Qiu's Neiyi recipe inhibited the MAPK activation signaling pathway in adenomyosis mice. (a) Effects of Qiu's Neiyi recipe on the protein levels of ERK, p-ERK8, JNK, p-JNK, p38, and p-p38 in the uterus tissue. (b–g) Statistical analysis of the effects on ERK, p-ERK8, JNK, p-JNK, p38, and p-p38 expressions in the uterus tissue. L-Qiu's Neiyi recipe: a low dose of Qiu's Neiyi recipe; M-Qiu's Neiyi recipe: a middle dose of Qiu's Neiyi recipe; H-Qiu's Neiyi recipe: a high dose of Qiu's Neiyi recipe. $\left(\bar{\chi }\pm s,n=3\right)$, ΔΔ*P* < 0.01 vs. control group,  ^*∗*^*P* < 0.05,  ^*∗∗*^*P* < 0.01 vs. model group.

Coincident with the qRT-PCR results, the protein expression of ERK, p-ERK, JNK, p-JNK, p38, and p-p38 were significantly increased in the tamoxifen-induced adenomyosis mice (*P* < 0.01). Danazol and middle and high doses of Qiu treatment decreased the expression of p-ERK, JNK, p-JNK, p38, and p-p38 significantly (*P* < 0.05). And the inhibition of Qiu on the activation of the MAPK signaling pathway was in a dose-dependent manner (Figure 6).

## 4. Discussion

The use of TCM for disease treatment is considered to have mild adverse reactions and to be relatively cost-efficient [21]. According to these strengths, TCM has been used more frequently worldwide. Qiu was designed based on the syndrome typing of TCM and followed the rules for drug synergism and compatibility [8]. The protective roles of Qiu and the active herbal in it for the treatment of adenomyosis have been confirmed clinically [8–10, 22, 23]. In this study, it was found that Qiu could attenuate the development of tamoxifen-induced adenomyosis in mice in a dose-dependent manner, and this may be related to the regulation of inflammatory MAPK signaling.

Adenomyosis is a chronic inflammatory disease. Its inflammatory pathogenesis is also involved in the abnormal immune responses, which can cause cellular and humoral immune abnormalities [24]. The systemic and local immune changes have been observed in women affected by adenomyosis, accompanied by the elevated levels of IL-6, IL-1*β*, IFN-*α*, TNF-*α*, and IFN-*γ*, and the coexistence of changes in inflammatory and anti-inflammatory signals [25]. The thymus and spleen are important immune organs in animals. They are responsible for the immune cell aggregation and immune response activation [26,27]. Therefore, the weight of the spleen and thymus can, to some extent, indicate the number of immune cells in the immune organ, thus indirectly reflecting the immune status of the body [28]. In this study, the endometrial glands, interstitial hyperplasia, and uterus weight were increased, while the spleen index and thymus index were decreased in adenomyosis mice. However, these symptoms significantly improved after being treated with Qiu. It attenuated the development of endometriosis‐like lesions, decreased the uterine weight, and increased the spleen and thymus index in adenomyosis mice. The result indicated that Qiu partly recovered the abnormal immune responses of adenomyosis.

Inflammatory cytokines or cell infiltration is usually observed in the pathology of adenomyosis [12]. IL-6 and IL-8 have been reported to promote the inflammatory pathological state of adenomyosis [29]. Inflammatory mediators could promote angiogenesis and interact with sensory neurons to induce the pain signal [30,31]. Due to the different states and locations of the disease, the thresholds of pain vary widely [32]. Li et al. [33] found that the increased production of proinflammatory mediators (IL-1*β*, IL-6, and TNF-*α*) can cause hyperalgesia and dysmenorrhea by inducing neuronal receptor activation. Although the studies about Qiu on adenomyosis-related inflammation are rare, the research studies on the herbs in Qiu also provides clues to its anti-inflammatory effect. *Epimedium brevicornu* Maxim. ethanol extract could exhibit an anti-inflammatory effect by inhibiting the production of several proinflammatory mediators, including NO, TNF-*α*, IL-1*β*, and IL-6 in LPS-induced peritonitis in vivo and in vitro [34]. *Morinda officinalis* How. is reported to exert an anti-inflammatory effect in rheumatoid arthritis, which mainly contributes to the iridoids in it [35]. In this study, the level of IL-1*β*, IL-6 and TNF-*α* were increased in serum and uterine tissue of mice with adenomyosis compared to the controls, this funding was consist with the aforementioned results reported by others. More importantly, Qiu treatment remarkably abolished the inflammatory cytokine elevation in adenomyosis model mice, accompanied by an improvement of the adenomyosis symptoms. Based on the aforementioned studies of herbs or compounds in Qiu with anti-inflammatory effects, we indicated that our funding also confirmed the anti-inflammatory potential of Qiu in adenomyosis, which contributed to its therapeutic effect on adenomyosis.

Meanwhile, the activation of proinflammatory mediators is critically associated with the regulation of inflammatory pathways in adenomyosis. The MAPK/ERK signaling pathway is proven to be activated in endometriosis and plays a key role in the inflammatory process and development of adenomyosis [17,18]. The proliferation of uterine smooth muscle cells is found to be increased in women with adenomyosis, accompanied by the activation of the MAPK/ERK cell-signaling pathway [3]. Ectopic endometrial cells are found to exhibit a hyperproliferative phenotype through ROS-related activation of the MAPK/ERK and PI3K/mTOR/AKT pathways in endometriosis [36]. Thus, the inhibition of the ERK/MAPK signaling pathway can repress the development of ectopic endometrial tissues in a rat model of endometriosis [37]. A bioactive component, 3,4-dihydroxyphenylethyl alcohol glycoside from *Sargentodoxa cuneata,* a herb of Qiu, has been reported to suppress the production of proinflammatory cytokines and the activation of the NF-*κ*B, STAT3, and p38 MAPK in an acute lung injury model of mice and MH-S cells [38]. Indirectly, this suggests the suppression ability of Qiu on the MAPK/ERK pathway. In the present study, we demonstrated that the development of adenomyosis in mice was associated with the activation of the MAPK/ERK pathway, and the mRNA and protein expressions of p-ERK, p-JNK, and p-p38 were increased. And this activation was related to the elevated levels of IL-1*β*, IL-6, and TNF-*α* in serum and uterine tissue of adenomyosis mice. More importantly, the activated MAPK/ERK pathway could be suppressed by Qiu in a dose-dependent manner, the levels of inflammatory mediators as well as the disease severity were also decreased in our study.

In conclusion, our study showed that Qiu treatment reduced the myometrial infiltration, attenuated relative organ indexes, and decreased the levels of inflammatory factors in mice with adenomyosis. And this effect of Qiu on adenomyosis was associated with the inhibition of the MAPK/ERK pathway. However, the therapeutic effect of Qiu for adenomyosis remains not thoroughly studied. Therefore, further research for Qiu is required to comprehensively detect the potential benefits on adenomyosis.

## Figures and Tables

**Table 1:** Primer sequence of the genes for qRT-PCR analysis.

Gene	Forward primer (5′–3′)	Reverse primer (5′–3′)
Mouse ERP	GGAGCAGCCTTAGTCCTGTC	TATGCAAGCGGAGTTCAGCA
Mouse JNK	CACCTTAAATCCTGCCCACG	ATGCACTGTGGGACTTCAGG
Mouse P38	TGGCCCTGCCTTTACCATATC	CAAACACATCCGTGCTCTGC
Mouse IL-1*β*	AAGGGGACATTAGGCAGCAC	ATGAAAGACCTCAGTGCGGG
Mouse TNF-*α*	TGTCTACTCCTCAGAGCCCC	GACCCGTAGGGCGATTACAG
Mouse IL-6	TCCGGAGAGGAGACTTCACA	CATAACGCACTAGGTTTGCCG
Mouse *β*-actin	TCTTTGCAGCTCCTTCGTTG	TCCTTCTGACCCATTCCCAC

## Data Availability

All data generated or analyzed during this study are included in this article.
